# A Common Minimal Motif for the Ligands of HLA-B*27 Class I Molecules

**DOI:** 10.1371/journal.pone.0106772

**Published:** 2014-09-30

**Authors:** Alejandro Barriga, Elena Lorente, Carolina Johnstone, Carmen Mir, Margarita del Val, Daniel López

**Affiliations:** 1 Centro Nacional de Microbiología, Instituto de Salud Carlos III, Majadahonda, Madrid, Spain; 2 Centro de Biología Molecular Severo Ochoa, CSIC/Universidad Autónoma de Madrid, Madrid, Spain; Albert Einstein Institute for Research and Education, Brazil

## Abstract

CD8^+^ T cells identify and kill infected cells through the specific recognition of short viral antigens bound to human major histocompatibility complex (HLA) class I molecules. The colossal number of polymorphisms in HLA molecules makes it essential to characterize the antigen-presenting properties common to large HLA families or supertypes. In this context, the HLA-B*27 family comprising at least 100 different alleles, some of them widely distributed in the human population, is involved in the cellular immune response against pathogens and also associated to autoimmune spondyloarthritis being thus a relevant target of study. To this end, HLA binding assays performed using nine HLA-B*2705-restricted ligands endogenously processed and presented in virus-infected cells revealed a common minimal peptide motif for efficient binding to the HLA-B*27 family. The motif was independently confirmed using four unrelated peptides. This experimental approach, which could be easily transferred to other HLA class I families and supertypes, has implications for the validation of new bioinformatics tools in the functional clustering of HLA molecules, for the identification of antiviral cytotoxic T lymphocyte responses, and for future vaccine development.

## Introduction

The proteolytic degradation of newly synthesized pathogen proteins in the cytosol through the combined actions of proteasomes and different peptidases continuously generates peptides of typically 8 to 11 residues long, and these fragments were translocated to the endoplasmic reticulum (ER) lumen through a transporter associated with antigen-processing (TAP) molecules. These short peptides are subsequently assembled with nascent human leukocyte antigen (HLA) class I heavy chain and β_2_-microglobulin molecules [1]. This assembly likely occurs through the interaction of the lateral chains of anchor residues at position 2 (P2) and the C-terminus (PΩ) of the antigenic peptide [2], [3], and these chains are inserted deeply into specific pockets of the antigen recognition groove of the HLA class I molecule [4], [5]. The stable HLA/peptide complexes are eventually exported to the cell membrane and presented for cytotoxic T lymphocyte (CTL) recognition [6]. The detection of pathogen peptides by specific T cell receptors results in the killing of pathogen-infected cells.

HLA class I is the largest polymorphic biological system described. More than 7,000 HLA class I alleles have been identified to date (Immuno Polymorphism Database, http://www.ebi.ac.uk/ipd), and classic HLA serologies have been largely divided into complex HLA gene families with increasing numbers of expressed protein subtypes. For example, to date, HLA-B*27 (a well-studied HLA class I family) comprises at least 100 different alleles. Although the presence of Arg at P2 is necessary for HLA-B*27 ligands (SYFPEITHI Database [3]), only a partial overlapping of the peptide repertoire has been observed in different HLA-B*27 subtypes [7]. Individual HLA-B*27 subtypes could present or not specific ArgP2-containing peptides, or the same ligand could bind to different HLA-B*27 subtypes with a broad range of affinity values [8]. Thus, the existence of HLA-B*27 ligands with additional binding motifs for presentation by all or most of the different HLA-B*27 subtypes remains unknown. To address this question, the binding affinity of a homogeneous set of nine naturally processed viral HLA-B*2705 ligands with different sequences, identified using mass spectrometry analysis of complex HLA-bound peptide pools isolated from large amounts of Human respiratory syncytial virus (HRSV)-infected cells [9], was examined using seven phylogenetically and functionally different major HLA-B*27 subtypes [10], [11]. This analysis revealed a common minimal peptide motif for efficient binding to different HLA-B*27 subtypes.

## Materials and Methods

### HLA-B*27 cell lines and antibodies

RMA-S is a TAP-deficient murine cell line that expresses the mouse H-2^b^ haplotype [12]. Transfected RMA-S cell lines expressing HLA-B*2701 [13], -B*2702 [13], -B*2703 [14], -B*2704 [8], -B*2705 [15], -B*2706 [8], or -B*2709 [16] have been previously described (summarized in Figure S1). All cell lines were cultured in RPMI 1640 medium supplemented with 10% heat-inactivated fetal bovine serum and 50 µM β-mercaptoethanol. ME1, a monoclonal antibody (mAb) specific for HLA-B27, -B7, and -Bw22 [17] and goat anti-mouse IgG-FITC (AbD Serotec, Kidlington, UK) were used in this study.

### Synthetic peptides

The peptides were synthesized in a peptide synthesizer (model 433A; Applied Biosystems, Foster City, CA) and subsequently purified through reversed-phase HPLC. The molecular mass of the peptides was established using MALDI-TOF MS, and the peptide composition was determined through µLC-MS/MS.

### HLA/Peptide Stability Assays

The synthetic peptide CMV pp65_294-302_ (VAFTSHEHF, HLA-C*012-restricted)[18] was used as a negative control in complex stability assays. In addition, for some HLA-B*27 subtypes, the Flu NP peptide (SRYWAIRTR, HLA-B27-restricted) [19] was used as a positive control. The transfected RMA-S B*27 cell lines were incubated at 26°C for 16 h to promote the expression of empty HLA class I molecules (without antigenic peptide) at the cell membrane, as these molecules are stable at 26°C but not at 37°C. The cells were washed and incubated for 2 h at 26°C with various concentrations of peptide in medium without fetal bovine serum. The cells were maintained at 37°C for an additional 2 h to facilitate the internalization of empty MHC class I molecules. Subsequently, the cells were collected for flow cytometry to discriminate between bound or unbound peptides. MHC expression was measured using 100 µl of hybridoma culture supernatant containing the ME1 (anti-HLA-B27) mAb and the secondary antibody as previously described [20]. The data were acquired using a FACSCanto flow cytometer (BD Biosciences, San Jose, CA, USA) and analyzed using BD FACSDiva software version 6 (BD Bioscience). The cells alone exhibited peak fluorescence intensities similar to the background staining observed with secondary Ab alone. The fluorescence index was calculated as the ratio of the mean peak channel fluorescence of the sample to that of the control incubated without peptide. Peptide binding was expressed as the EC_50_, the molar concentration of the peptide at 50% of the maximum fluorescence obtained at a concentration range of 0.01–200 µM.

### Statistical analysis

Unpaired Student's *t* test was used to analyze statistical significance. *P* values<0.05 were considered significant.

## Results

### HRSV specific ligands bind to the B*2705 molecule with a broad range of affinities

**Figure 1:**
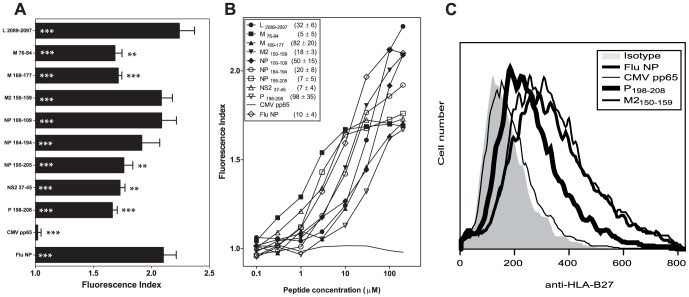
HLA-B*2705 stabilization with synthetic HRSV ligands. The stability of HLA-B*2705/peptide complexes on the cell surface of transfected RMA-S cells was measured through flow cytometry. Panel A: The indicated peptides were used at 200 µM. The CMV pp65 and Flu NP peptides were used as negative and positive controls, respectively. The mAb ME1 was used for staining. The results, calculated as fluorescence indexes, show the means ± SD of 4-5 independent experiments. Significant P values (***, p<0.001; **, p<0.01; *, p<0.05) versus negative or positive peptide controls are represented as white or black asterisks, respectively. Panel B: The titration curves for the indicated synthetic peptides with HLA-B*2705 are depicted. The results show the mean values obtained from three or four independent experiments. The calculated EC_50_ values (µM) (means ± SD) are shown in the legend of panel B. A representative experiment is depicted in panel C.

Nine HLA-B*2705-restricted ligands endogenously processed and presented in the HRSV-infected cells were previously identified (Table S1) [9]. To confirm that HLA-B*2705 is the Major Histocompatibility Complex (MHC) class I molecule responsible for the presentation of these ligands, MHC/peptide complex stability assays were performed using TAP-deficient RMA-S cells transfected with the HLA-B*2705 molecule. Four HRSV synthetic peptides (L _2089-2097_, M2 _150-159_, NP _100-109_ and NP _184-194_) showed stable numbers of HLA-peptide surface complexes similar to those of the well known HLA-B*2705 epitope from influenza virus (Figure 1A and Table 1). For the other five HRSV synthetic peptides (M _76-84_, M _169-177_, NP _195-205_, NS2 _37-45_ and P _198-208_), the induction of HLA-peptide surface complexes was somewhat lower than that of the positive control (Figure 1A and Table 1). In addition, the relative MHC class I affinity was determined for all HRSV peptides, according to the EC_50_ value. The peptides bound to HLA-B*2705 class I molecules at a broad range of EC_50_ values commonly observed among natural ligands: high (M _76-84_, M2 _150-159_, NP _184-194_, NP _195-205_ and NS2 _37-45_), medium (L _2089-2097_, and NP _100-109_) or low affinity (M _169-177_ and P _198-208_) (Figure 1B and Table 2). A representative experiment is shown in Figure 1C. These data confirm that all ligands detected in HRSV-infected cells were endogenously presented in association with the B*2705 molecule.

**Table 1:** Summary of HLA stabilization assays using synthetic ligands.

Peptide	Sequence	B*2705	B*2703	B*2704	B*2706	B*2701	B*2702	B*2709
L _2089-2097_	GRNEVFSNK	2.2±0.1^a^	1.8±0.1	1.5±0.2	1.7±0.1	1.0±0.1	1.2±0.1	1.6±0.1
M _76-84_	SRSALLAQM	1.7±0.1	1.8±0.2	1.8±0.1	2.6±0.4	1.6±0.1	1.3±0.1	1.7±0.1
M _169-177_	VRNKDLNTL	1.7±0.1	1.5±0.1	1.8±0.2	3.0±0.2	1.2±0.2	1.4±0.1	1.6±0.1
M2 _150-159_	KRLPADVLKK	2.1±0.1	1.7±0.1	1.8±0.1	1.5±0.1	1.0±0.1	1.4±0.1	1.6±0.1
NP _100-109_	HRQDINGKEM	2.1±0.1	1.8±0.1	1.7±0.2	2.3±0.1	1.2±0.1	1.3±0.1	1.5±0.1
NP _184-194_	RRANNVLKNEM	1.9±0.2	1.8±0.1	1.9±0.2	3.0±0.3	1.6±0.1	1.5±0.1	1.8±0.1
NP _195-205_	KRYKGLLPKDI	1.8±0.1	1.7±0.1	2.1±0.2	2.6±0.4	1.7±0.1	1.7±0.1	1.6±0.1
NS2 _37-45_	HRFIYLINH	1.7±0.1	1.9±0.1	1.5±0.1	2.0±0.1	1.1±0.2	1.5±0.1	1.5±0.1
P _198-208_	LRNEESEKMAK	1.7±0.1	1.3±0.1	1.1±0.1	1.2±0.1	1.0±0.1	1.0±0.1	1.5±0.1
CMV pp65	VAFTSHEHF	1.0±0.1	1.0±0.1	1.0±0.1	1.0±0.1	1.0±0.1	1.0±0.1	1.1±0.1
Flu NP	SRYWAIRTR	2.1±0.1	1.7±0.2	2.0±0.2	2.6±0.2	1.0±0.1	1.1±0.1	1.5±0.1

a Data are expressed as fluorescence index when peptides were used at 200 µM ± S.D. The results show the mean of three to five independent experiments. All data show significant P values versus the negative control CMV pp65 peptide, except the seven values underlined (see figures).

**Table 2:** Affinity values of HRSV ligands for different HLA-B27 subtypes.

Peptide	Sequence	B*2705	B*2703	B*2704	B*2706	B*2701	B*2702	B*2709
L _2089-2097_	GRNEVFSNK	**32±6** a	71±28	169±57	>200	-	>200	68±24
M _76-84_	SRSALLAQM	**5±5**	**6±3**	**15±6**	**1±1**	**15±11**	>200	**6±2**
M _169-177_	VRNKDLNTL	82±20	>200	**56±16**	**3±1**	>200	94±25	**19±7**
M2 _150-159_	KRLPADVLKK	**18±3**	78±10	73±9	>200	-	136±63	**22±9**
NP _100-109_	HRQDINGKEM	**50±15**	96±43	67±27	**3±2**	>200	132±53	**24±7**
NP _184-194_	RRANNVLKNEM	**20±8**	**60±29**	**19±10**	**2±1**	**39±9**	**43±27**	**7±1**
NP _195-205_	KRYKGLLPKDI	**7±5**	**20±11**	**16±3**	**1±1**	**8±1**	**9±1**	**7±2**
NS2 _37-45_	HRFIYLINH	**7±4**	**10±5**	78±33	**45±19**	-	77±14	**33±9**
P _198-208_	LRNEESEKMAK	98±35	>200	-	>200	-	-	86±12
HIV gp160 _500-508_	KRAVGIGAL	**12±3**	**14±7**	**24±8**	**1±1**	**17±3**	**16±6**	**8±1**
MV F _438-466_	RRYPDAVYL	**3±2**	**4±3**	**18±4**	**2±1**	**5±2**	**11±4**	**4±1**
Flu PB1 _238-246_	RRAIATPGM	**3±2**	**2±1**	**9±1**	**2±1**	**5±1**	**12±4**	**2±1**
RRA_7_I	RRAAAAAAAI	**4±3**	**7±5**	**7±2**	**2±1**	**7±2**	**28±16**	**2±1**
Flu NP	SRYWAIRTR	**10±4**	**23±3**	**19±9**	**18±4**	-	>200	**26±3**

a Data are expressed as EC_50_ (µM) ± S.D and are the mean of three to five independent experiments. Affinity values indicating intermediate affinity peptides (20<EC_50_≤60 µM) are marked in bold and values indicating high affinity peptides (EC_50_≤20 µM) are also underlined. – indicates no statistical difference in fluorescence index compared to the negative control.

### The His59Tyr change in the A pocket induces a moderate decrease in the binding of HRSV-specific ligands to HLA-B*27

**Figure 2:**
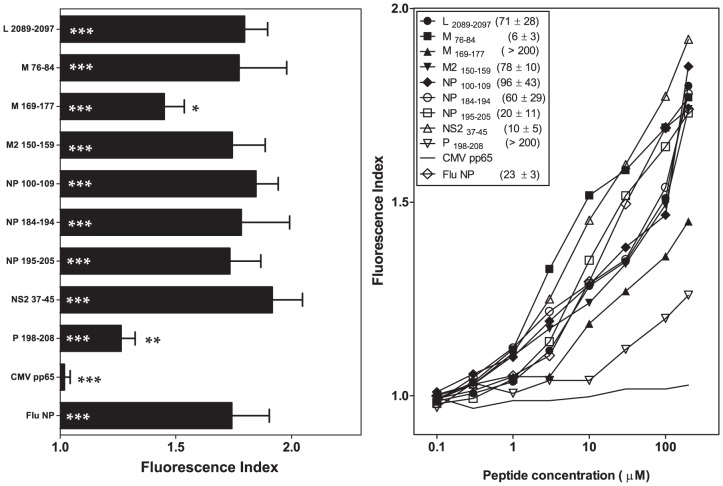
HLA-B*2703 stabilization with synthetic HRSV ligands. The stability of HLA-B*2703/peptide complexes on the cell surface of transfected RMA-S cells was measured using flow cytometry. The results shown in left and right panels are depicted as in Fig. 1A and 2B, respectively, showing the mean values obtained from three or four independent experiments.

The B*2703 molecule differs from the prototypical subtype HLA-B*2705, reflecting a single amino acid change at heavy-chain residue 59, which is responsible for anchoring the N-terminus of the peptide within the A pocket of the class I molecule (Figure S1). The analysis of MHC/peptide complex stability assays using RMA-S cells transfected with the HLA-B*2703 molecule showed either no effect (M _76-84_, NP _195-205_ and NS2 _37-45_) for three HRSV synthetic peptides or a moderate decrease in the EC_50_ value (L _2089-2097_, M _169-177_, M2 _150-159_, NP _100-109_, NP _184-194_ and P _198-208_) for six HRSV synthetic peptides in response to the His59Tyr change (Figure 2 and Tables 1 and 2).

### Basic, but not nonpolar, C-terminal residues reduce binding to the B*2704 molecule of viral B*2705-restricted ligands

**Figure 3:**
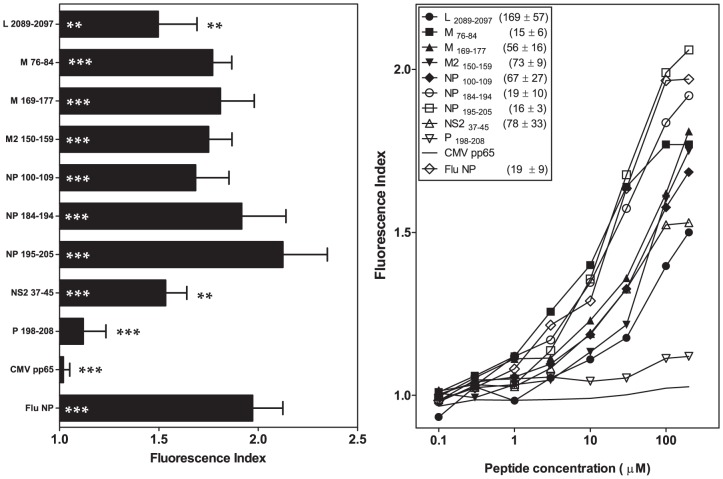
HLA-B*2704 stabilization with synthetic HRSV ligands. The stability of HLA-B*2704/peptide complexes on the cell surface of transfected RMA-S cells was measured through flow cytometry. The results shown in left and right panels are depicted as in Fig. 1A and 2B, respectively, showing the mean values obtained from three or four independent experiments.

In the binding groove, the B*2704 subtype differs from the B*2705 molecule, reflecting two amino acid changes at residues 77 (Asp to Ser) and 152 (Val to Glu) located in the F and E pockets, respectively (Figure S1). The analysis of peptide binding to HLA-B*2704 in transfected RMA-S cells showed almost no effect with the five HRSV synthetic peptides harboring nonpolar amino acids at the PΩ residue (M _76-84_, M _169-177_, NP _100-109_, NP _184-194_, and NP _195-205_; Tables 1 and 2, and Figure 3) compared with HLA-B*2705 affinity. In contrast, according to the B*2704-EC_50_, three HRSV ligands with basic PΩ residues showed a moderately decreased affinity to B*2704 compared with B*2705 (L _2089-2097_, M2 _150-159_, and NS2 _37-45_). Moreover, P _198-208_, the low affinity ligand for the B*2705 molecule, did not bind to the B*2704 subtype (Figure 3 and Tables 1 and 2).

### HRSV B*2705-restricted ligands with nonpolar PΩ residues exhibit high-affinity binding to B*2706

**Figure 4:**
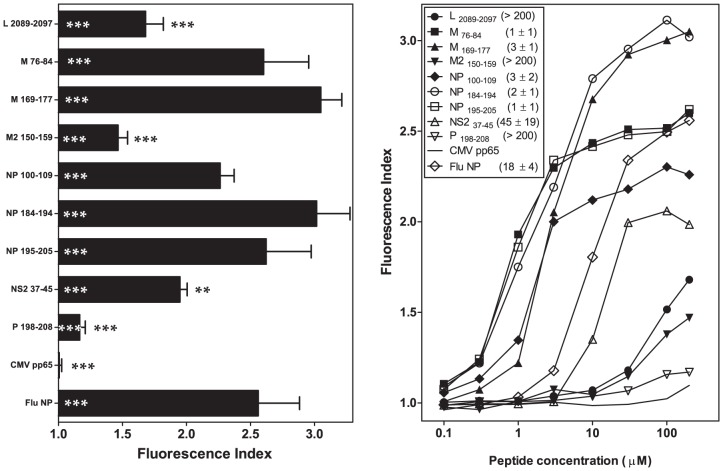
HLA-B*2706 stabilization with synthetic HRSV ligands. The stability of HLA-B*2706/peptide complexes on the cell surface of transfected RMA-S cells was measured through flow cytometry. The results shown in left and right panels are depicted as in Fig. 1A and 2B, respectively, showing the mean values obtained from three or four independent experiments.

B*2704 and B*2706 differ at two amino acids, His114Asp and Asp116Tyr, located in the same strand of the β-pleated sheet floor of the peptide binding site of HLA-B*27 (Figure S1). These changes highly stabilized (in the range of µM) the binding of the five HRSV ligands harboring nonpolar PΩ residues (M _76-84_, M _169-177_, NP _100-109_, NP _184-194_, and NP _195-205_) to B*2706 (Figure 4 and Tables 1 and 2). In contrast, a moderate increase (NS2 _37-45_ and P _198-208_) or decrease (L _2089-2097_, and M2 _150-159_) in affinity to B*2706 was observed for HRSV peptides containing basic amino acids in the PΩ residue compared with binding to B*2704. Thus, nonpolar PΩ residues could be used as additional strong auxiliary anchor motifs in B*2706 ligands.

### High-affinity binding to B*2709 of viral ligands with nonpolar PΩ residues

**Figure 5:**
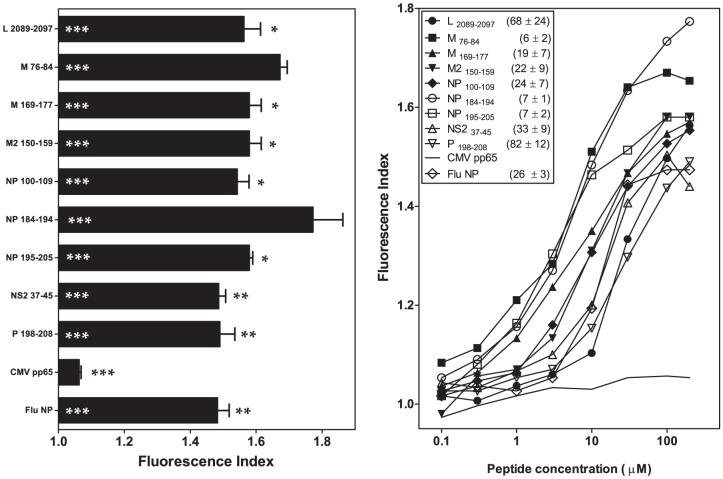
HLA-B*2709 stabilization with synthetic HRSV ligands. The stability of HLA-B*2709/peptide complexes on the cell surface of transfected RMA-S cells was measured through flow cytometry. The results shown in left and right panels are depicted as in Fig. 1A and 2B, respectively, showing the mean values obtained from three or four independent experiments.

B*2709 differs from B*2705 by a single amino acid change, Asp116His, located in a strand of the β-pleated sheet floor of the peptide binding site of HLA-B*27 (Figure S1). This change stabilized (M _76-84_, NP _184-194_, and NP _195-205_) or increased (M _169-177_, and NP _100-109_) the binding of the five HRSV ligands harboring nonpolar PΩ residues to B*2709 compared with binding to the prototypical subtype B*2705 (Figure 5 and Tables 1 and 2). In contrast, a moderate decrease in affinity was observed for two HRSV peptides containing basic amino acids in the PΩ residue (L _2089-2097_, and NS2 _37-45_) compared with binding to B*2705, except with M2 _150-159_ ligand where a basic amino terminal residue could compensate the loss of interaction in the F pocket. Thus, nonpolar PΩ residues are sufficient for efficient binding to the B*2709 class I molecule.

### Nonpolar C-terminal residues in high affinity B*2705-restricted viral ligands preserve the binding affinity to the B*2701 molecule

**Figure 6:**
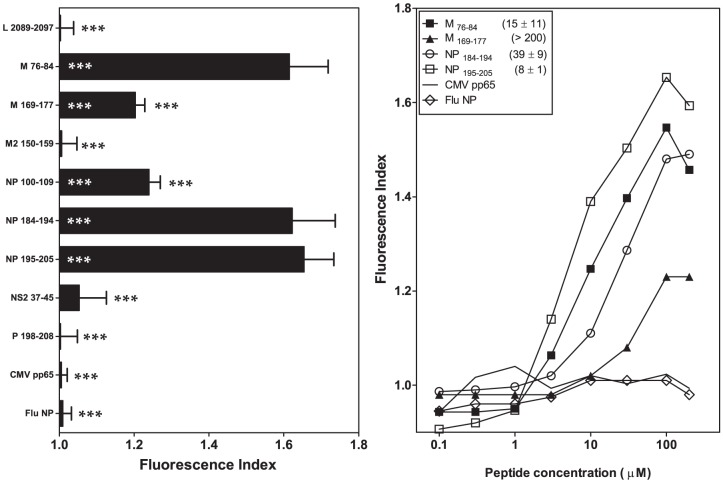
HLA-B*2701 stabilization with synthetic HRSV ligands. The stability of HLA-B*2701/peptide complexes on the cell surface of transfected RMA-S cells was measured through flow cytometry. The results shown in left and right panels are depicted as in Fig. 1A and 2B, respectively, showing the mean values obtained from three or four independent experiments.

B*2701 differs from B*2705, reflecting three amino acid changes, Asp74Tyr, Asp77Asn, and Leu81Ala, located in the C/F cavity of the HLA-B*27 peptide binding site (Figure S1). These changes destabilized the binding of all HRSV peptides except those ligands with nonpolar amino acids in the PΩ residue, which exhibited high affinity for the B*2705 molecule (M _76-84_, NP _184-194_, and NP _195-205_) (Figure 6 and Tables 1 and 2). Thus, nonpolar PΩ residues are necessary, but not sufficient, for efficient binding to the B*2701 class I molecule.

### Hydrophobic C-terminal residues, such as Leu or Ile, preserve the binding to the B*2702 molecule of HRSV B*2705-restricted ligands

**Figure 7:**
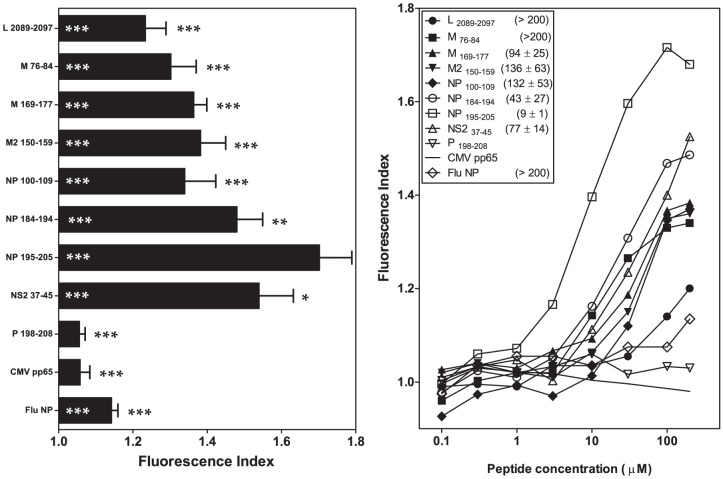
HLA-B*2702 stabilization with synthetic HRSV ligands. The stability of HLA-B*2702/peptide complexes on the cell surface of transfected RMA-S cells was measured through flow cytometry. The results shown in left and right panels are depicted as in Fig. 1A and 2B, respectively, showing the mean values obtained from three or four independent experiments.

The B*2702 subtype differs from the B*2705 molecule, reflecting three amino acid changes at positions 77 (Asp to Asn), 80 (Thr to Ile) and 81 (Asp to Tyr), located in the F pocket (Figure S1). Only the two HRSV peptides with Leu/Ile PΩ residues, M _169-177_ and NP _195-205_, similarly stabilized the binding to both B*2705 and B*2702 molecules (Figure 7 and Tables 1 and 2). In contrast, the binding affinity to B*2702 was moderately decreased for the NP_184-194_ peptide, and strongly reduced for the other six viral ligands (Tables 1 and 2). Thus, hydrophobic PΩ residues, such as Leu/Ile, serve as additional auxiliary anchor motifs for B*2702-binding.

### A common minimal motif for ligands of the seven different HLA-B*27 subtypes

Table 3 summarizes the HLA-B*27-binding patterns of the different HRSV B*2705-restricted ligands used in this study (Table S1). Most of the viral ligands bound to various HLA-B*27 subtypes, despite amino acid differences in relevant residues that contribute to the antigen binding site; however, only two of these ligands (NP _184-194_, and NP _195-205_) efficiently bound to all seven HLA-B*27 subtypes studied. Both ligands contain basic amino acids in the N-terminus and a large and hydrophobic lateral chain in the C-terminal residues. This structure establishes the Arg/Lys-Arg-X_n_-Ile/Met sequence as the common minimal peptide motif for efficient binding to seven different HLA-B*27 subtypes. To test this prediction three different peptides, HIV gp160 _500-508_ [21], MV F _438-446_ [22], and Influenza virus PB1 _238-246_ [23], harboring the identified minimal peptide motif (basic residue at P1 and large hydrophobic aliphatic residue at PΩ), were studied. Interestingly, MHC/peptide complex stability assays showed efficient binding of these synthetic peptides to all seven HLA-B*27 subtypes tested (Tables 2 and 3). In addition, a synthetic peptide with only the common minimal peptide motif (RRAAAAAAAI) efficiently bound to all seven HLA-B*27 subtypes analyzed (Tables 2 and 3). Conversely, the exchange for Ala of the Met residue at CΩ position in both NP _184-194_, and NP _195-205_ peptides abolished the interaction with B*2701 subtype (Table 4). Also, this monosubstitution significantly decreased the binding of A11-NP _195-205_ peptide to B*2702 subtype (Table 4). Moreover the exchange for Ala of the Arg residue at P1 position in NP _184-194_ peptide considerably decreased the binding to B*2703 subtype (Table 4).

**Table 3:** Summary of the relative affinity of HRSV ligands for different HLA-B27 subtypes.

Peptide	Sequence^a^	B*2705	B*2703	B*2704	B*2706	B*2701	B*2702	B*2709
L _2089-2097_	GRNEVFSNK	+++ b	++	++	+	-	+	++
M _76-84_	SRSALLAQM	++++	++++	++++	++++	++++	+	++++
M _169-177_	VRNKDLNTL	++	+	+++	++++	+	++	++++
M2 _150-159_	KRLPADVLKK	++++	++	++	+	-	++	+++
NP _100-109_	HRQDINGKEM	+++	++	++	++++	+	++	+++
NP _184-194_	RRANNVLKNEM	++++	+++	++++	++++	+++	+++	++++
NP _195-205_	KRYKGLLPKDI	++++	++++	++++	++++	++++	++++	++++
NS2 _37-45_	HRFIYLINH	++++	++++	++	+++	-	++	+++
P _198-208_	LRNEESEKMAK	++	+	-	+	-	-	++
HIV gp160 _500-508_	KRAVGIGAL	++++	++++	+++	++++	++++	++++	++++
MV F _438-466_	RRYPDAVYL	++++	++++	++++	++++	++++	++++	++++
Flu PB1 _238-246_	RRAIATPGM	++++	++++	++++	++++	++++	++++	++++
RRA_7_I	RRAAAAAAAI	++++	++++	++++	++++	++++	+++	++++
Flu NP	SRYWAIRTR	++++	+++	++++	++++	-	+	+++

a The new HLA-B27 anchor motifs for efficient HLA binding are underlined.

b +, ++, +++ and ++++ indicate EC_50_ values>200 µM, 200-61 µM, 60-20 µM, and <20 µM, respectively. – indicates no statistical difference in fluorescence index compared to the negative control. All positive EC_50_ data show significant P values (P<0.01) versus the negative control.

**Table 4:** HLA stabilization assay with monosubstituted Ala analogs of HRSV NP _184-194_ and NP _195-205_ synthetic ligands.

Peptide	Sequence	B*2705	B*2703	B*2704	B*2706	B*2701	B*2702	B*2709
NP _184-194_	RRANNVLKNEM	1.9±0.2 a	1.8±0.2	1.9±0.2	3.0±0.3	1.6±0.2	1.5±0.1	1.8±0.1
A1-NP _184-194_	ARANNVLKNEM	2.4±0.3	**1.4±0.1**	2.2±0.2	3.0±0.3	2.0±0.1	2.0±0.3	1.8±0.1
A11-NP _184-194_	RRANNVLKNEA	2.1±0.1	1.7±0.2	2.0±0.2	2.7±0.2	1.1±0.1	1.7±0.1	1.8±0.1
NP _195-205_	KRYKGLLPKDI	1.8±0.1	1.7±0.1	2.1±0.2	2.6±0.3	1.7±0.1	1.7±0.1	1.6±0.1
A1-NP _195-205_	ARYKGLLPKDI	2.4±0.3	2.1±0.2	2.2±0.3	2.7±0.3	1.9±0.1	1.8±0.1	1.8±0.2
A11-NP _195-205_	KRYKGLLPKDA	2.2±0.2	1.9±0.1	1.9±0.3	2.5±0.2	1.0±0.1	**1.4±0.1**	1.6±0.2
CMV pp65	VAFTSHEHF	1.0±0.1	1.0±0.1	1.0±0.1	1.0±0.1	1.0±0.1	1.0±0.1	1.1±0.1

a Data are expressed as fluorescence index when peptides were used at 200 µM ± S.D. The results show the mean of three to five independent experiments. All data show significant P values (P<0.01) versus the negative control CMV pp65, except the two values underlined. In addition, the fluorescence index of A1-NP _184-194_ with B*2703 subtype or A11-NP _195-205_ peptide with B*2702 subtype (marked in bold) also shows significant P values (P<0.01) versus either the negative control or the NP _184-194_ and NP _195-205_ peptides, respectively.

Compared with the affinity of the nine HRSV ligands for B*2705, 7 ligands exhibited decreased affinity for B*2701 and B*2702, 6 ligands showed decreased affinity for B*2703, 4 ligands showed reduced affinity for B*2704 and B*2706, and only 3 ligands exhibited decreased affinity for B*2709 (summarized in Table 3). These data do not correlate with either the amino acid differences between subtypes or the individual interactions in the respective pockets, indicating compensatory effects of changes in some residues. These effects were more evident with the B*2706 subtype, showing increased affinity for some HRSV ligands with nonpolar PΩ residues compared with the B*2705 subtype. Thus, based on the affinity patterns with HLA-B*27 subtypes, a functional relationship between B*2705 and the other subtypes could be established as B*2705>B*2709>B*2706>B*2704>B*2703>B*2701 = B*2702.

## Discussion

Using large scale mass spectrometry analysis, an extensive knowledge of HLA-B*2705 ligandome (with approximately 2,000 peptides identified) has been reported [7], [24]. In contrast, few endogenous natural ligands have been identified in other HLA-B*27 subtypes: 32, 49, 38 and 50 from B*2703, B*2704, B*2706 and B*2709, respectively, and only 8 and 15 from B*2701 and B*2701, respectively (summarized in [7] and SYFPEITHI database [3]). Except for the ArgP2 residue, no additional anchor or auxiliary anchor motifs were identified in these studies. Thus, in the present study the Arg/Lys-Arg-X_n_-Ile/Leu/Met sequence was identified as the minimal common peptide motif for efficient binding to the seven major, phylogenetically (Figure S2) and functionally different HLA-B*27 subtypes [10], [11], thereby establishing the criteria to analyze the influence of the HLA-B*27 polymorphism on pathogen peptide presentation and T cell epitope predictions for the rational design of vaccines to treat large HLA families.

Extensive analyses of alloreactive T cell responses from HLA-B*27-negative individuals against B*2702 [25], B*2703 [10], B*2704 [26], B*2705 [10], and B*2709 [27] subtypes at the clonal level to assess T cell epitope sharing among HLA-B*27 subtypes have been previously described. These studies defined the functional relationship as B*2705>B*2709>B*2703>B*2702>B*2701>B*2704>B*2706, reflecting amino acid differences between subtypes. However, in contrast to self-restricted pathogen recognition in normal cellular immune responses, the allogenic anti-B*27 CTL clones recognized both polymorphic allo-MHC residues and self-derived peptides [14]. Thus, the direct contribution of these polymorphic HLA-B*27 residues to the CTL epitope could disguise both cross-presenting antigen peptide properties and the degree of functional relationship between the different HLA-B*27 subtypes. As the antigen processing and presentation machinery is similar, if not identical, in all cell lines expressing the different HLA-B*27 subtypes, the nine HLA-B*2705-restricted ligands endogenously processed and presented in the HRSV-infected cells might also be presented by the diverse HLA-B*27 subtypes according to affinity. Thus, the functional relationship between subtypes established with the HRSV ligands through affinity assays in the current report could be a better approximation to determine the antigen peptide-presenting properties of HLA-B*27 class I molecules, and this information could be applicable to the rational design of vaccines.

Few studies have analyzed antigen binding and/or presentation of the same viral ligands to different HLA-B27 subtypes. Four EBV ligands (EBNA3C _258-266_, RRIYDLIEL; EBNA3B _243-253_, RRARSLSAERY; LMP2 _236-244_, RRRWRRLTV; EBNA3C _343-351_, FRKAQIQGL) in B*2702, B*2704, and B*2705 subtypes were tested, and only the EBNA3C _258-266_ ligand tended to be immunodominant and was recognized in the context of all three B27 subtypes studied, whereas the LMP _236-244_ ligand was only recognized associated to B*2704 [28]. In contrast, in another study, the LMP _236-244_ ligand was recognized by one of four CTL clones in the context of five HLA-B*27 (B*2702, B*2704, B*2705, B*2706 and B*2709) subtypes analyzed [29]. In addition two ligands, HIV gag _265-279_ (KRWIILGNKIVRMYC) and Flu NP _380-393_ (ELRSRYWAIRTRSG), were presented by both B*2702 and B*2705 subtypes [30]. Only one of these six viral epitopes harbors the minimal peptide motif for the efficient binding described in the current study, and this peptide was restricted by the three HLA-B27 subtypes analyzed (B*2702, B*2704 and B*2705). However, only an endogenous ligand derived from human histone H3.3 has been previously described for binding to B*2701 [13], B*2702 [31], B*2703 [32], B*2704 [33], B*2705 [34], B*2706 [33] and B*2709 [16] subtypes. This ligand, containing the RRYQKSTEL sequence, also harbors a basic amino acid in the N-terminal residue and a large and hydrophobic lateral chain in C-terminal residue, consistent with the motif defined in the present study.

Thus, studies examining different HLA class I families and supertypes are needed to determine the conserved anchor or auxiliary motifs common to these HLA clusters and validate new bioinformatics tools for the functional clustering of MHC molecules [35]. These data are also relevant for the identification of antiviral cytotoxic T lymphocyte responses and vaccine development.

## Supporting Information

Figure S1 Scheme of the polymorphisms in each HLA-B*27 subtype.
(PDF)

Figure S2 Phylogenetic tree of the HLA-B*27 subtypes **[36], [37].**
(PDF)

Table S1 **Summary of HRSV ligands.**
(PDF)
