# Supplementary material for: A Common Minimal Motif for the Ligands of HLA-B*27 Class I Molecules
Source: PLoS One. 2014 Sep 30;9(9):e106772. doi: 10.1371/journal.pone.0106772 (PMC4182091; doi:10.1371/journal.pone.0106772)
Supplement: Figure S1 — Scheme of the polymorphisms in each HLA-B*27 subtype. (PDF) [file pone.0106772.s001.pdf]

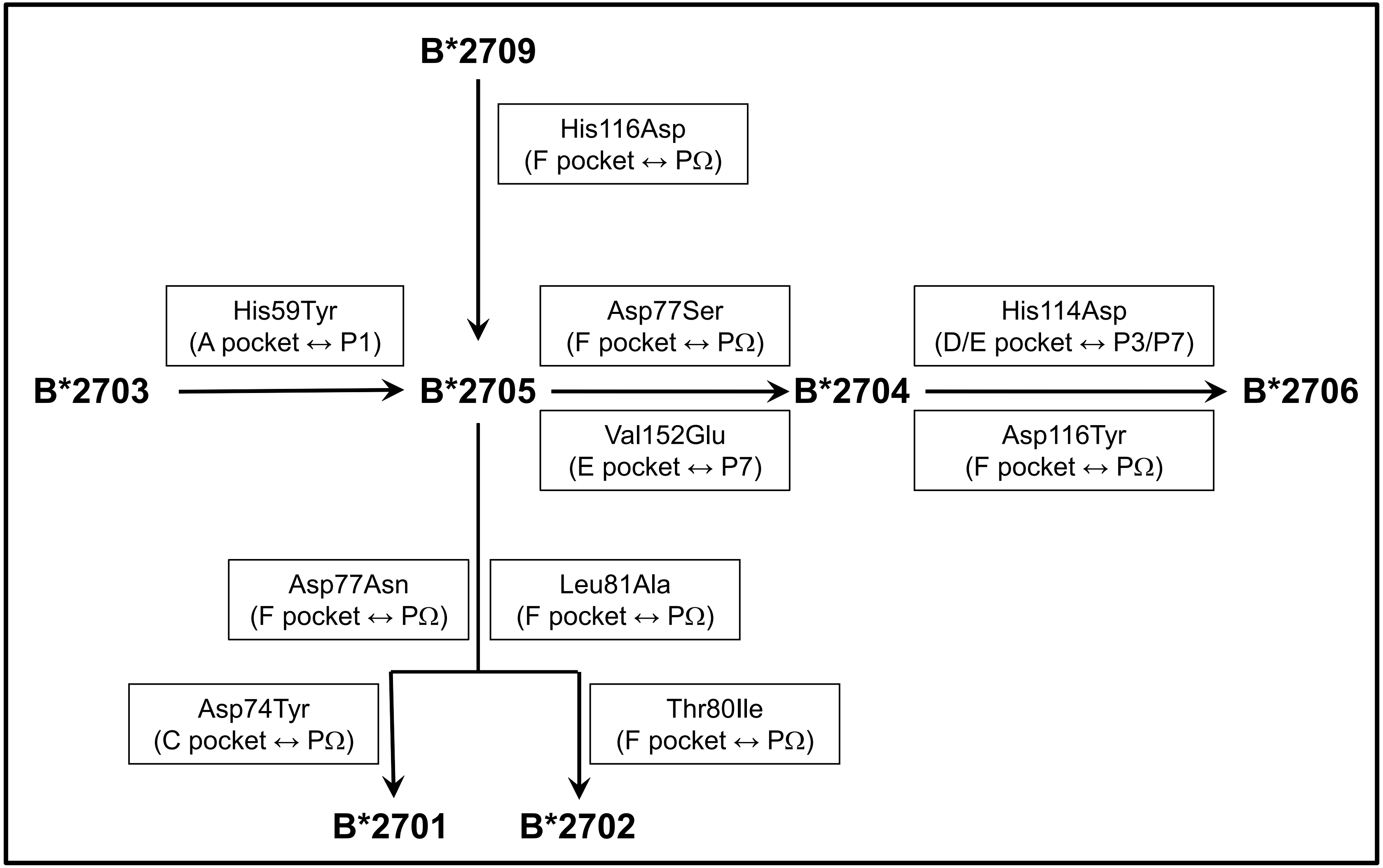

Barriga et al. Supplemental Figure 1

Figure S1. Scheme of the polymorphisms in each HLA-B*27 subtype

The changes in the subtypes analyzed in this study are indicated according to the three-letter code and position. The location and interaction of the respective residues in HLA-B*27 pockets (A to F) with the respective antigenic peptide residues (P1 to PΩ) are indicated.
