# Supplementary material for: A Common Minimal Motif for the Ligands of HLA-B*27 Class I Molecules
Source: PLoS One. 2014 Sep 30;9(9):e106772. doi: 10.1371/journal.pone.0106772 (PMC4182091; doi:10.1371/journal.pone.0106772)
Supplement: Figure S2 — Phylogenetic tree of the HLA-B*27 subtypes [36], [37]. (PDF) [file pone.0106772.s002.pdf]

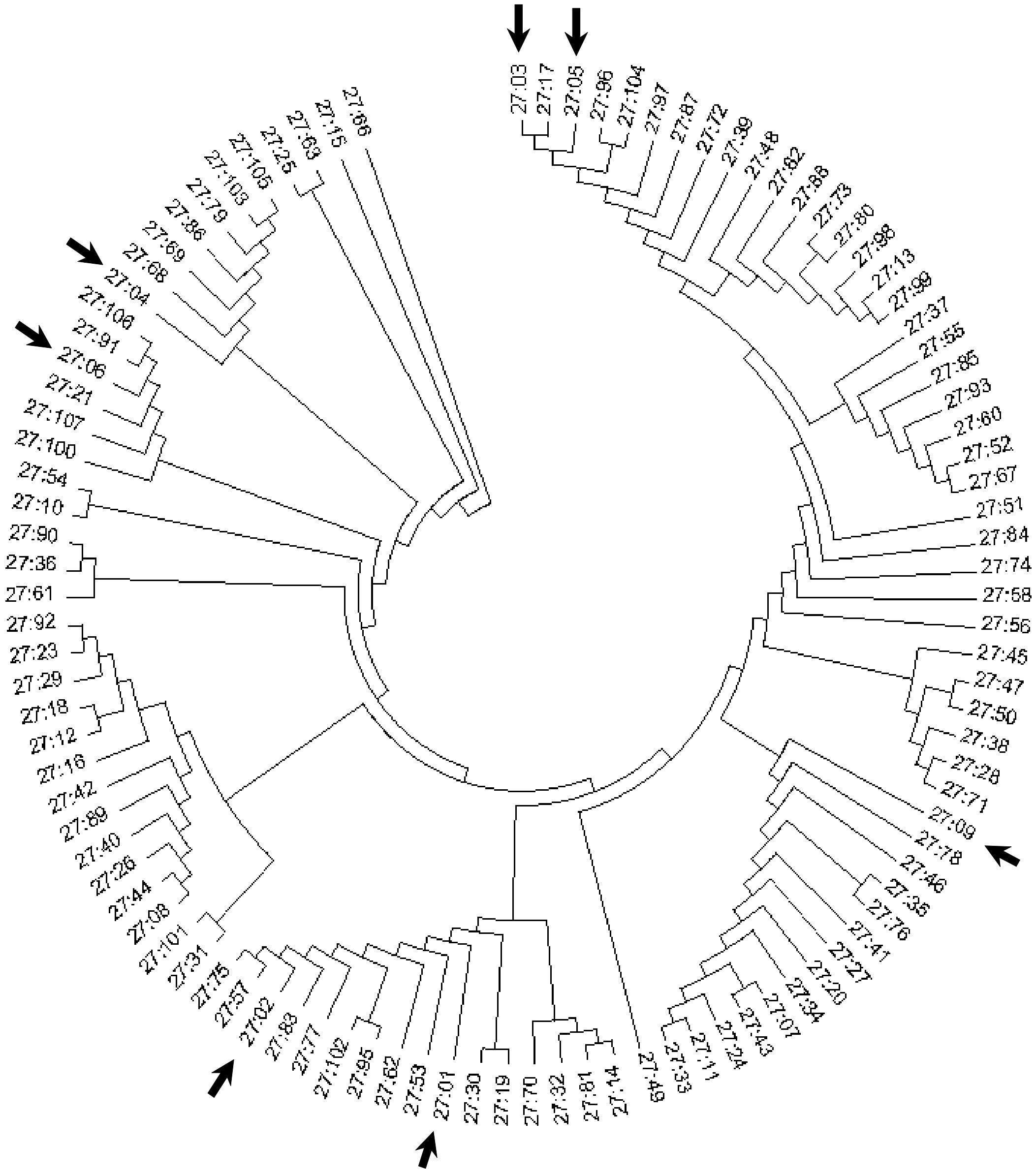

Barriga et al. Figure S2

Figure S2. Phylogenetic tree of the HLA-B*27 subtypes

Phylogenetic and molecular evolutionary analyses using the α1 and α2 antigen-presenting domains (180 residues) of 101 HLA-B*27 subtypes obtained from the IMGT/HLA Database [36] were performed using MEGA version 5.2.2, the neighbor-joining method and the Poisson model [37]. The subtypes examined in this study are indicated with an arrow.
