# Supplementary material for: A Common Minimal Motif for the Ligands of HLA-B*27 Class I Molecules
Source: PLoS One. 2014 Sep 30;9(9):e106772. doi: 10.1371/journal.pone.0106772 (PMC4182091; doi:10.1371/journal.pone.0106772)
Supplement: Table S1 — Summary of HRSV ligands. (PDF) [file pone.0106772.s003.pdf]

Table S1

Summary of B*2705 HRSV ligands derived from HRSV proteins

Ligand [a]	Protein	Position	Sequence
L $_{2089-2097}$	Polymerase	2089-2097	GRNEVFSNK
M $_{76-84}$	Matrix	76-84	SRSALLAQM
M $_{169-177}$	Matrix	169-177	VRNKDLNTL
M2 $_{150-159}$	Matrix 2-22k	150-159	KRLPADVLKK
NP $_{100-109}$	Nucleoprotein	100-109	HRQDINGKEM
NP $_{184-194}$	Nucleoprotein	184-194	RRANNVLKNEM
NP $_{195-205}$	Nucleoprotein	195-205	KRYKGLLPKDI
NS2 $_{37-45}$	Non-structural protein 2	37-45	HRFIYLINH
P $_{198-208}$	Phosphoprotein	198-208	LRNEESEKMAK

[a] The ligands were identified in Long strain of HRSV [9].
